# Preparation of a Novel Carbon Nano Coating on Carbon Fiber Surface Based on Plasma Electrolysis Effect

**DOI:** 10.3390/ma18174093

**Published:** 2025-09-01

**Authors:** Xin He, Qian Zhou, Maoyuan Li, Dongqin Li, Chiyuhao Huang, Xiaolin Wei, Weiwei Chen

**Affiliations:** 1 Department of Materials Science and Engineering, Beijing Institute of Technology, Beijing 100081, China; xin_he0205@163.com (X.H.); 17812162775@163.com (Q.Z.); 15210581440@163.com (D.L.); cyh_huang@163.com (C.H.); 3120235757@bit.edu.cn (X.W.); 2 Beijing System Design Institute of Electro-Mechanic Engineering, Beijing 100854, China; maoyuan_li@163.com

**Keywords:** cathode plasma electrolytic deposition, carbon fiber, carbon coating, oxidation resistance, tensile performance after heating

## Abstract

In this study, glucose is used as the source of C; through cathode plasma electrolytic deposition technology, a carbon nano coating is prepared on the surface of carbon fiber. The carbon coating is analyzed using scanning electron microscopy, transmission electron microscopy, and X-ray photoelectron spectroscopy to investigate the effect of pH on the microstructure of the carbon coating on the surface of carbon fiber. At the same time, the oxidation resistance of the coating and the changes in the tensile properties of carbon fiber after high-temperature heat treatment were also investigated. The results showed that reducing the pH value can improve the microstructure of the carbon coating, and the best performance of the carbon coating sample was obtained at pH = 3. The initial oxidation temperature and oxidation termination temperature increased by 156 °C and 110 °C, respectively, compared to the treated carbon fiber but without coating, and the tensile property remains a high value (2740 MPa) after high-temperature heat treatment.

## 1. Introduction

Due to its higher specific strength and specific stiffness than other materials, its high temperature stability, and its lower density, carbon fiber (CF) enforced carbon bonded composite materials (C/C for short) have been widely used in aerospace, especially in flights and thrusters [1,2,3,4,5]. Though it can keep stable in a temperature of 2000 °C without O_2_, it is oxidized rapidly in an environment with O_2_ as long as the temperature is higher than 400 °C, which exactly influences the extension of C/C [6,7]. Many efforts have been put in. Up until now, there have been two main methods used by researchers for the improvement of anti-oxidation performance, which are matrix doping and surface coating. In the field of coatings, several methods are used, including packing cementation (PC), chemical vapor deposition (CVD), plasma spraying (PS), and so on [8,9,10]. Qiang et al. successfully used CVD to prepare SiCNWs-SiC coating [11]. The bending strength of SiC-coated C/C composites gains an increase of 25.28% attributed to the incorporation of SiC nanowires. Furthermore, in wind tunnel tests, the SiCNWs-SiC coated C/C composites exhibited exceptional erosion resistance, with a mere 5.2% weight loss after enduring 53 h of erosion at 1873 K and undergoing eight thermal shock cycles from room temperature to 1873 K. ZrB_2_-HfB_2_-SiC-TaSi_2_ quaternary coating was prepared by Wang et al., with atmospheric pressure plasma spraying used [12]. In this coating system, TaSi_2_ not only produces SiO_2_ instead of B_2_O_3_ to fill the coating holes but also produces the more stable intermediate oxides TaZr_2.75_O_8_ and Hf_6_Ta_2_O_17_, with Ta_2_O_5_, ZrO_2_, and HfO_2_. These substances play the role of nailing cracks in the coating, increasing the glass viscosity, inhibiting the oxide phase transition, and effectively improving the oxygen resistance of the coating. In the field of doping, similarly, many efforts have been put in by numerous researchers. Due to the efforts of Li and others, the C/C-ZrC-SiC composites are successfully prepared using the precursor impregnation and pyrolysis (PIP) method with ZrCl_4_, TEOS, and FA as raw materials [13]. The flexural strength of the composite materials obtained reached 193.2 MPa, which is 58.78% higher than that of the composite materials prepared using organic zirconium-containing polymers (PCZ) and polycarbosilane (PCS) precursors [13]. The composites exhibited a superior ablation resistance, with a mass ablation rate of 0.10 mg/s and a linear ablation rate of 0.03 μm/s. Meanwhile, Makarov et al. successfully prepared composite fibers using the solid-state dissolution of cellulose in N-methylmorpholine-N-oxide with added tetraethoxysilane (TEOS) [14]. It was shown that adding TEOS to the cellulose matrix increased the fiber carbon residue up to 16% during heat treatment. And, there are bonds formed between Si and C during their high-temperature treatment according to IR spectra of the composite cellulose fibers. Additionally, it was found that the elasticity modulus and relative elongation of the CFs were double those of the C-SiC composite fibers, and the strength characteristics of the composite fibers decreased by 45% as compared with CFs produced from cellulose fiber [14]. Above all, it can be seen that both coating and doping do improve the antioxidant performance of C/C. However, the adhesion between the coating and the substrate, as well as the thermal compatibility between the coating and the substrate after heating, are both difficult issues. As for doping, the quality of CF will be greatly reduced in the doping process because of the high temperature. These issues hinder C/C’s further development.

CF refers to high-strength and high modulus fibers with a carbon content of over 90%. As the reinforcement of C/C, CF has a great influence on the performance of C/C. Additionally, in numerous coating systems, carbon coating has a thermal expansion coefficient similar to that of C/C, because of which stress cracks can be effectively prevented [15]. As for methods to modify CF, electrophoretic deposition, CVD, and hydrothermal carbonization methods (HTC) are used more widely. With the efforts of Li and his partners, vertically aligned carbon nanotube/carbon fiber (VACNT/CF) hybrids are prepared by electrophoretic deposition (EPD) under mild conditions [16]. And, the contact angle and the interfacial shear strength (IFSS) are significantly improved by 48.3% and 58.1%, respectively, compared to that of original CF. Due to the efforts of Zou et al., monolithic CNTs@CF fibers consisting of a 3D highly porous CNT sponge layer with a macroscopic thickness (up to several millimeters), which is directly grown on a single CF, are fabricated [17]. And, the high sponge–CF interfacial strength, owing to the presence of a thin transitional layer, completely inhibits the CF slippage from the matrix upon fracture in CNTs@CF fiber–epoxy composites. Song et al. successfully prepared functional hydrothermal carbonization coatings (HTCCs) on carbon fibers in a carbon fiber braid via a facile hydrothermal carbonization process of widely sourced carbohydrates to obtain a robust sorbent. Similarly, smooth, uniform, and controllable hydrothermal carbon coatings (HTCCs) have been successfully constructed on carbon fibers through the efficient hydrothermal transformation approach from renewable carbohydrates by Fang et al. [18]. In their study, the thickness of HTCC could be effectively tailored from nanoscale to micro-scale by just adjusting the concentration of carbohydrate solutions [18].

However, these methods may have high energy consumption (CVD), have significant damage to CF (CVD), or take a long time to successfully deposit (EPD and HTC). To a certain degree, all these disadvantages restrict the further development of these methods. Benefiting from the progress of technology, the appearance of cathode plasma electrolytic deposition technology gives researchers the potential to settle these two problems through the depositing of carbon coating on the surface of the CF through cathode plasma electrolytic deposition (CPED for short). Due to its convenience, high efficiency, and energy efficiency, CPED receives the favor of many researchers. So far, it is quite developed in the preparation of metal coatings, alloy coatings, and ceramic coatings on the surfaces of metal or alloy matrix [19,20,21,22,23]. Meanwhile, researchers also achieved the deposition of ceramic coating on fibers, including CF, quartz fiber, and TiNb fiber [24,25,26,27,28,29,30].

In recent years, the carbonization of biomass has gradually attracted the attention of researchers because of its renewability, and successes have been made by researchers [31,32]. Furthermore, it is well known to us all that glucose is a monosaccharide that is non-toxic, harmless, inexpensive, and easily soluble in water. Carbon coating has been successfully prepared using glucose and an HTC method [18,33]. And, there are also examples using CPED, but not glucose [34,35,36]. Therefore, in this study, we attempt to deposit carbon coating on the surface of CF through CPED with glucose as the source of carbon; less energy is used while coating, and the process is more environmentally friendly in electrolyte processing.

## 2. Experiment

### 2.1. Materials Preparation

As is shown in Table 1, CF is produced by HSCARBONFIBRE (Danyang, China), which is named HF40S. Table 2 shows parts of the performances of HF40S. Glucose is produced by KANGMEI (Chengdu, China) and is medicinal grade. Hydrochloric acid is produced by Sinopharm Chemical Reagent Co., Ltd. (Beijing, China). As for the device for deposition, it is self-developed.

### 2.2. Experiment Procedure

**Figure 1:**
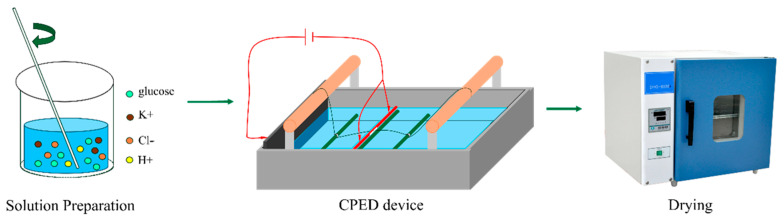
Experimental route.

Figure 1 shows the experiment route. Before depositing, a mixed solution of potassium chloride and glucose was prepared using deionized water, with a concentration of 20 g/L for KCl and a concentration of 17.5 wt% for glucose. HCl was used to adjust the pH from 6 to 2. Then, the voltage was set to 220 V and the CF movement speed to 2.8 cm/s, and the plasma electrolytic deposition of carbon coating was started on the surface of the CF. Table 3 shows the specific parameters while depositing. After that, coated CF were dried in the atmosphere of air at 100 °C for 1 h. At this point, the sample preparation process came to an end.

### 2.3. Characterization Techniques

The observation of CF sample morphology was mainly achieved by SEM, using a Japanese JSM 7200F (JEOL Ltd., Tokyo, Japan) scanning electron microscope. The distribution of elements on the coating surface was observed by EDS, using Oxford X-Max (Oxford Instruments, Abingdon, UK).

To observe more details of the coating, especially the possible crystalline phase of C, TEM was performed and a FEI Talos F200X (FEI Company, Hillsboro, OR, USA) transmission electron microscope was used.

Using a LabRAM HR Evolution confocal laser light spectrometer (Horiba Scientific, Kyoto, Japan), Raman spectroscopy testing was conducted, with a 532 nm light source used. At least five points from each bundle sample were taken and pulled to focus on the center position of CF in the Raman microscope for testing.

XPS was achieved by PHI QUANTERA-Ⅱ SXM (Physical Electronics, Chanhassen, MN, USA) photoelectron spectroscopy with a 1486.6 eV AlKa source. Signals ranging from 0 to 1100 eV were collected to form an energy spectrum.

### 2.4. Performance Testing

To know about the performance of coated CF, using the results from DSC-Mettler, according to GB/T 23442-2009 [37], the initial oxidation temperature of each sample is counted. In this study, tensile performance is also discussed. With a heat treatment of 600 °C for 20 min, each sample’s tensile strength is obtained according to GB/T 3362-2017 [38].

## 3. Results and Discussion

### 3.1. Micro Morphology of Carbon Coating

**Figure 2:**
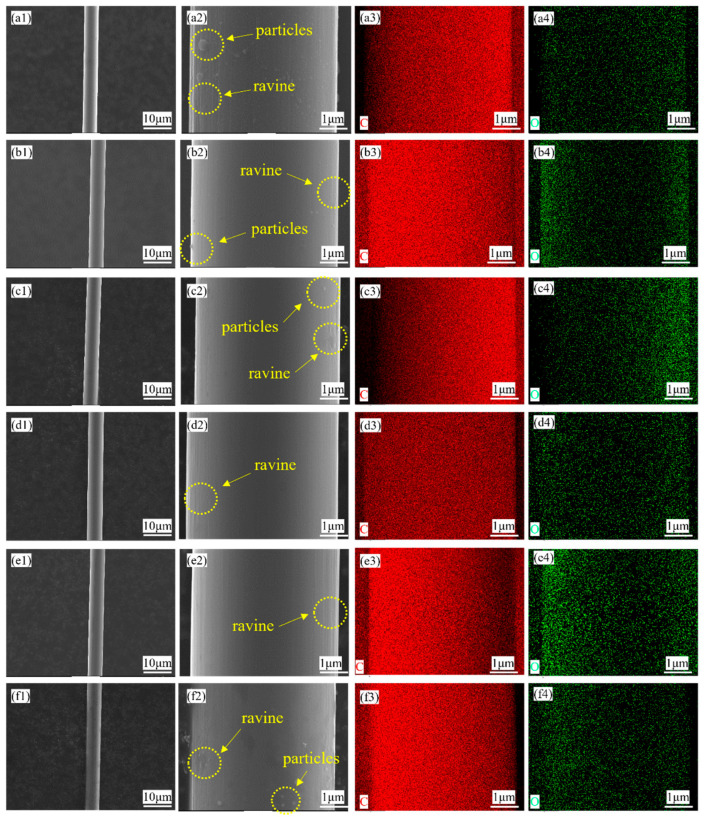
The SEM and EDS results of each carbon fiber: (**a1**–**a4**) bare; (**b1**–**b4**) pH = 6; (**c1**–**c4**) pH = 5; (**d1**–**d4**) pH = 4; (**e1**–**e4**) pH = 3; (**f1**–**f4**) pH = 2.

Figure 2 shows the surface morphology of the CF, including samples coated with different pH levels and one sample without Sizing agent after plasma electrolysis processing. Figure 2(a2) shows that there are some ravines along the fiber and particles attached to the fiber, which exactly shows the original surface of the CF. In Figure 2(b2,c2,d2), ravines and particles still exist. But, the particles are becoming smaller and smaller. As for the ravines, they seem to be more and more misty. In Figure 2(e2), ravines seem to be filled gradually, and the surface of the CF is more and more smooth. Additionally, the number of particles on the surface also becomes less and less. As a result of this, there seems to be no particles in Figure 2(e2). These two phenomena both point to the existence of the coating on the CF. However, in Figure 2(f2), particles appear again. It seems to be that the coating has the best micro morphology in the condition of pH = 3. After observation under the scanning electron microscope, EDS was conducted. Figure 2 also shows the result of the mapping scan, containing the energy spectrum of different elements and their own atomic percentage. Obviously, there is a little oxygen on the surface of these samples. Furthermore, C and O both distribute uniformly. Both surface morphology and elemental distribution show the coating’s uniformity.

**Figure 3:**
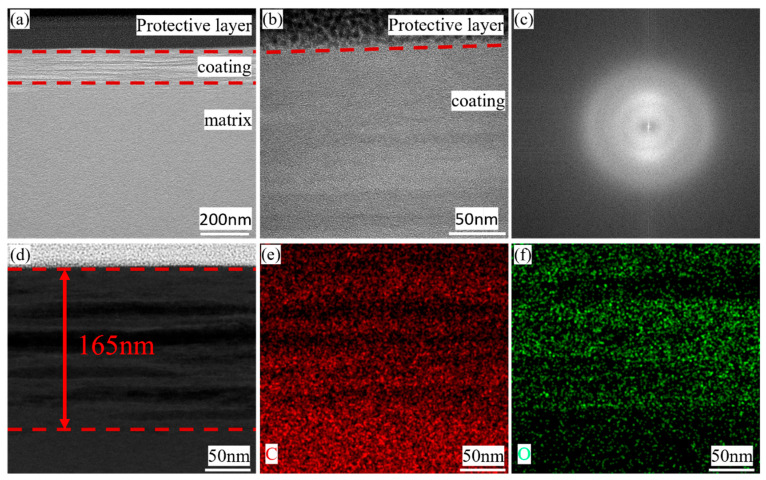
The TEM results of the sample: (**a**) FIB sample in high power; (**b**) FIB sample in high resolution mode; (**c**) FTT result; (**d**) The thickness of the coating; (**e**) C; (**f**) O.

To learn more about the microstructure of the coating, one sample with coating was observed under the transmission electron microscope. Before doing that, this sample was sliced into one piece using FIB technology. The details of the progress while cutting the sample are shown in the Supplementary Materials, Figure S1. Figure 3 shows the details of the protective layer of Pt, the coating, and a part of the CF. It is worth noticing that the protective Pt layer was used here to minimize surface damage caused by processing. In Figure 3a, it is found that there is a thin layer that is exactly located between the protective layer and the CF. Additionally, it is found that there is a wrinkle-like structure in the longitudinal section of the coating, seeming to be a defect or some different phases. The answer will be revealed later. Figure 3b, the photo with a high resolution, indicates that the form of the existence of carbon in the coating is the same as the CF, and both are amorphous. Figure 3c is the FTT result of 3b, showing that this coating is made of amorphous carbon. Figure 3d shows that this coating has a thickness of 160 nm or so. Considering that the scanning range of SEM is larger than that of TEM, mapping was conducted in TEM similarly, with the details shown in Figure 3e,f. According to this, it is also obvious that there is a difference in the distribution of oxygen. It can be seen that O lies in the wrinkle-like areas more, because of which these wrinkles are the results of glucose’s different degrees of restoration rather than defects in coatings. Above all, results supported by TEM strongly prove the existence of carbon coating.

### 3.2. Phase Analysis

**Figure 4:**
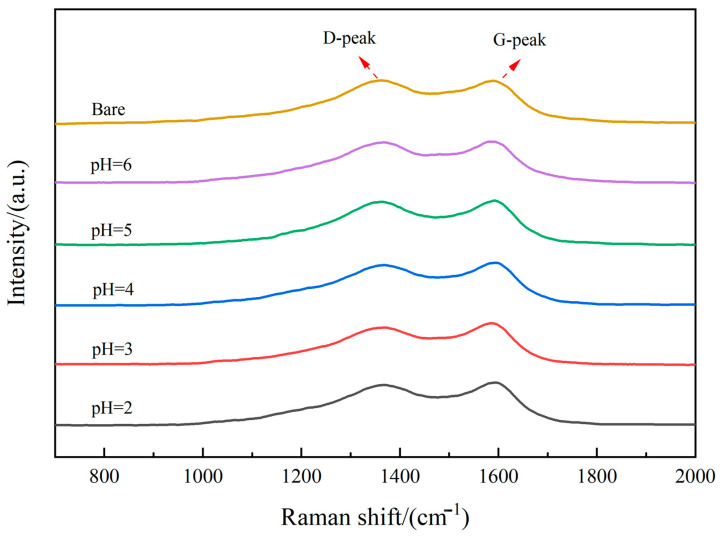
Raman spectrum of each sample.

Raman spectroscopy was performed to assess the graphitization degree of the coatings, with results presented in Figure 4. Distinct D-peaks (disorder-induced) and G-peaks (graphite-related) were observed at approximately 1350 cm^−1^ and 1580 cm^−1^, respectively. These peak positions are characteristic of carbon materials and align with the established literature [18,33,39,40,41]. The intensity ratio (I_D_/I_G_) serves as an indicator of the graphitization level, with calculated values detailed in Table 4. All coated samples exhibited I_D_/I_G_ ratios exceeding 0.8, confirming the predominantly disordered, amorphous nature of the carbon coating. Notably, a progressive decrease in the I_D_/I_G_ ratio was observed from unmodified fibers to coated samples, and further from pH = 6 (1.008) to pH = 3 (0.901), indicating an enhanced graphitic ordering as pH decreased within this range. However, this trend reversed at pH = 2, where the I_D_/I_G_ ratio increased instead of decreasing, establishing a consistent pattern where further acidification beyond pH = 3 detrimentally impacts graphitization.

**Figure 5:**
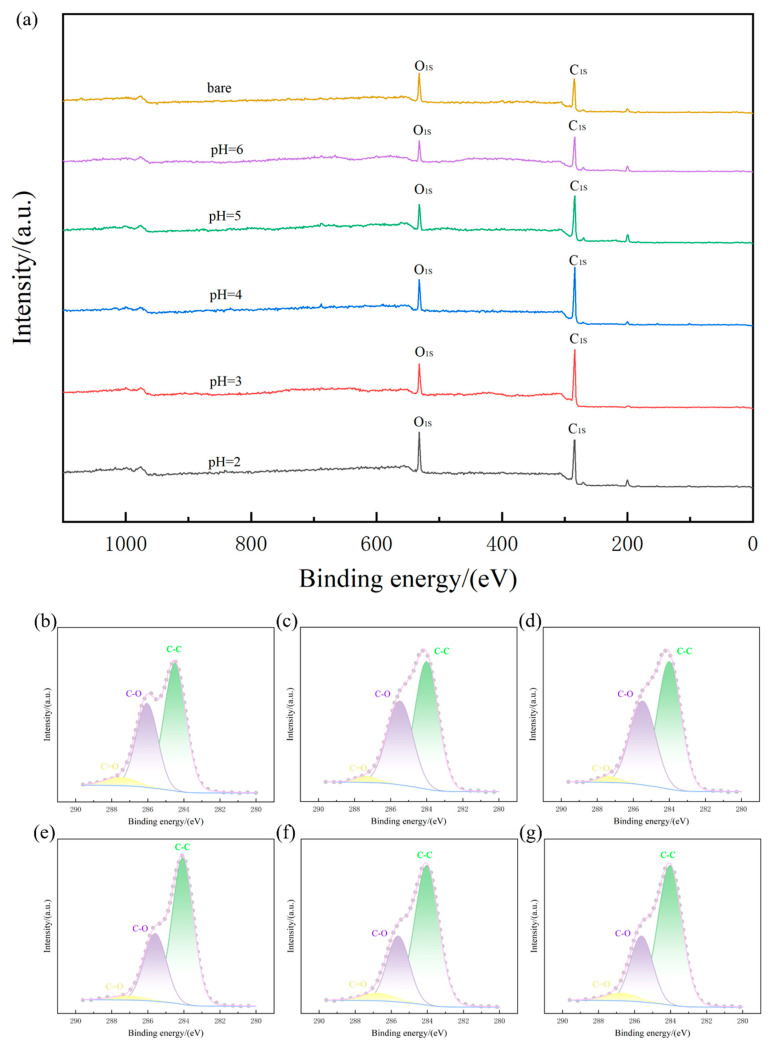
XPS of each sample: (**a**) full spectrum diagram; (**b**) C_1s_ peak of bare sample; (**c**) C_1s_ peak of sample in pH = 6; (**d**) C_1s_ peak of sample in pH = 5; (**e**) C_1s_ peak of sample in pH = 4; (**f**) C_1s_ peak of sample in pH = 3; (**g**) C_1s_ peak of sample in pH = 2.

XPS was conducted to investigate the chemical state of carbon in the coatings, with results shown in Figure 5a. The intensity of the C_1s_ peak progressively increased from uncoated carbon fibers to coated samples, and further increased as the deposition pH decreased from 7 to 3. However, the sample coated at pH = 2 exhibited slightly reduced C_1s_ intensity compared to pH = 3. Quantitative analysis of atomic percentages (detailed in Table 5) revealed a consistent trend: oxygen content decreased while carbon content increased as pH lowered from neutral to pH = 3. This inverse relationship indicates the enhanced reduction efficiency of glucose-derived carbon at moderately acidic conditions. Notably, the reversal of this trend at pH = 2—where oxygen content increased relative to pH = 3—demonstrates that excessively low pH negatively impacts the carbon deposition process, confirming that extreme acidity is suboptimal for coating formation.

The preceding discussion relied on the full XPS spectrum. Further analysis was performed through deconvolution of the C_1s_ peak, detailed in Figure 5b–g. The C_1s_ peak was resolved into three distinct components: a primary peak at 284.3 eV corresponding to C–C bonds, a secondary peak at 285.7 eV representing C–O bonds, and a minor peak at 287.0 eV attributed to C=O bonds. The dominant intensity of the C–C peak aligns with the carbon–oxygen atomic concentration data presented in Table 5. Existing literature indicates that the C–C and C–O signals originate primarily from the carbon fiber substrate, while the C=O component results from incomplete glucose decomposition during processing [33,39]. Comparative analysis across all coated samples reveals a consistent shift toward lower binding energies in the C_1s_ spectrum. This shift indicates that the carbon within the deposited coating exhibits a lower valence state compared to the substrate carbon fiber.

### 3.3. Performance of the Coating Testing

**Figure 6:**
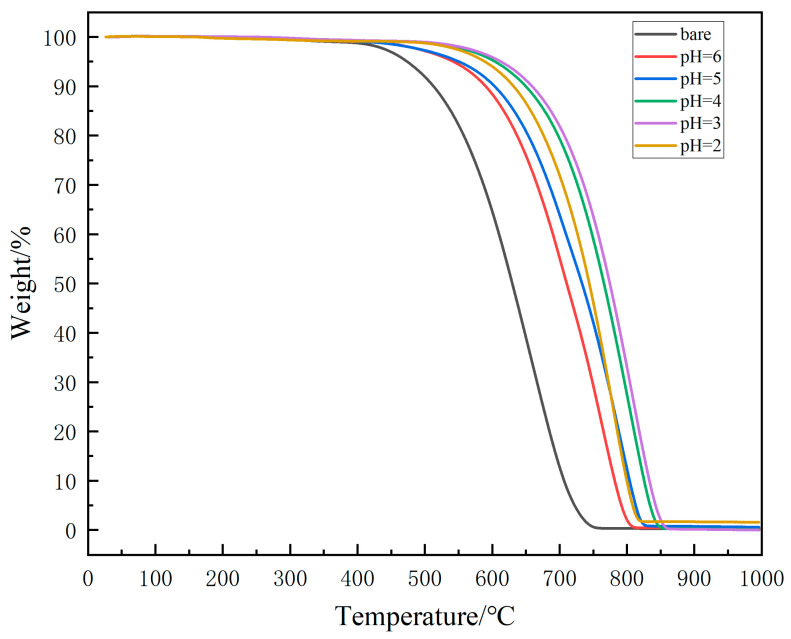
Thermogravimetric curve of each sample.

To learn about the performance of the coating, TG was performed to test the coating’s oxidation resistance. Figure 6 shows the six samples’ TG curves. According to GB/T 23442-2009 [37], the initial oxidation temperature was calculated, together with the complete weightlessness temperature of CF, are calculated and listed in Table 6. Both Figure 6 and Table 6 suggest that samples with coating have a better oxidation resistance performance. From Table 6, it is clear that coated samples’ initial oxidation temperature increases at least 70 °C (sample pH = 6) and at most 156 °C (sample pH = 3). Similarly, oxidation resistance also shows enhancing until pH = 3, and then weakening.

**Figure 7:**
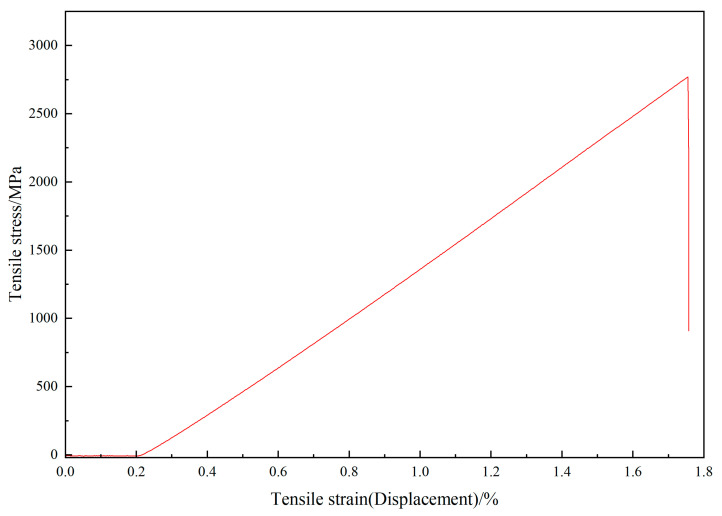
The relationship between tensile stress and tensile strain.

Having undergone a heat treatment of 600 °C for 20 min, the sample (pH = 3) remains at a tensile strength of 2740 MPa, while the bare sample remains minimally. This shows the excellent protective ability of this carbon coating to the CF. Figure 7 shows the relationship between tensile stress and tensile strain. It is obvious that it does not break until the tensile stress achieves 2740 MPa. Compared with Table 2, coating prepared at the pH value of 3 keeps the tensile strength of CF at 48.9% after the heat treatment in 600 °C for 20 min. This shows the protection effect of this carbon coating.

### 3.4. Analysis of Carbon Coating Deposition Mechanism

**Figure 8:**
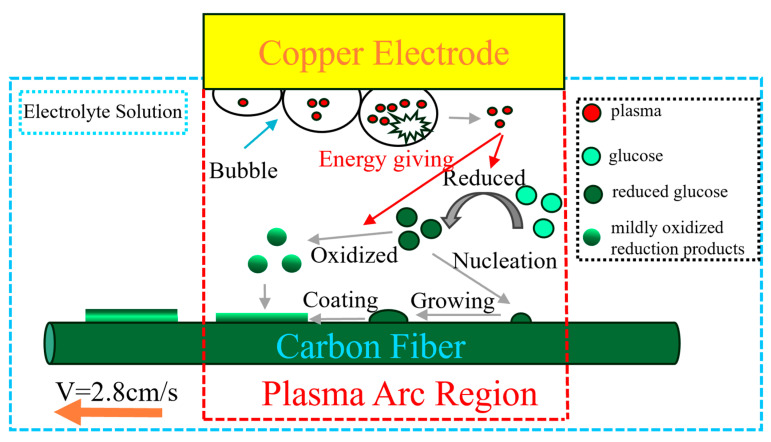
The deposition mechanism of the carbon coating.

Figure 8 illustrates the coating deposition process. Traditional glucose-derived carbon coating methods necessitate acidic solutions, prolonged heating, and extended reaction times. In this conventional approach, glucose undergoes sequential conversion to fructose and furan intermediates before nucleating and growing as carbon spheres on the fiber surface [42]. Conversely, cathode plasma electrolytic deposition (CPED) operates at ultra-high temperatures (thousands of degrees Celsius) with dramatically shorter reaction times (mere seconds in this study). The reduction in surface ravines observed during deposition indicates preferential nucleation near existing surface irregularities due to their enhanced surface area. The extreme thermal environment of CPED enables the instantaneous conversion of glucose into carbonaceous material that rapidly nucleates and grows directly onto the carbon fiber substrate. This efficient carbonization is evidenced by the low C–O and C=O peak intensities in XPS analysis. However, the intense plasma conditions simultaneously promote oxidation of the carbonaceous material. As the carbon fiber traverses the plasma zone, material near the arc center experiences heightened thermodynamic activity and deposits first, while material farther from the center avoids oxidation and deposits subsequently. This spatiotemporal deposition sequence creates an oxygen gradient within the coating cross-section, with higher oxygen content internally and lower externally. The extreme processing temperature also yields distinctive morphological characteristics: unlike traditional carbon sphere formations, the coating exhibits no discernible interface with the substrate (Figure 3a,b), consistent with the established research demonstrating that repeated thermal treatments eliminate coating–matrix interfaces.

## 4. Conclusions

This study successfully demonstrates a rapid and energy-efficient method for depositing a uniform amorphous carbon nano coating (~160 nm) on CF via cathode plasma electrolytic deposition (CPED) using a glucose electrolyte. The optimized coating (pH = 3) significantly enhances oxidation resistance, increasing the initial oxidation temperature by 156 °C (from 540 °C to 696 °C) while maintaining an excellent interfacial compatibility. Furthermore, this optimized coating (pH = 3) makes the tensile strength of the CF remain 2740 Mpa after the heat treatment of 600 °C for 20 min. Characterization reveals that the coating’s amorphous structure and oxygen-segregated lamellar features result from the plasma-driven instantaneous carbonization of glucose. This work provides a promising alternative to conventional coating techniques, with potential applications in high-temperature aerospace components. Further research should focus on scaling up the process and evaluating its long-term stability under cyclic thermal conditions.

## Figures and Tables

**Table 1:** Material specifications.

Name	Pureness/Model	Manufacturer
CF	HF40S	HSCARBONFIBRE
Glucose	CP	KANGMEI
KCl	99.5%	Zhan Cheng (Tianjin) Technology (Tianjin, China)
HCl	36.5%	Sinopharm Chemical Reagent Co., Ltd.
Deionized water	99.9%	-

**Table 2:** Partial characteristics table of HF40S.

Name	Linear/(g/km)	Tensile Strength/(MPa)	Tensile Modulus/(GPa)
Value	445	5600	295

**Table 3:** Deposition parameters.

Sample Number	Bare	pH = 6	pH = 5	pH = 4	pH = 3	pH = 2
Parameter						
Concentration of KCl	20	20	20	20	20	20
Concentration of glucose	0	17.5	17.5	17.5	17.5	17.5
Voltage/(V)	220	220	220	220	220	220
pH	7	6	5	4	3	2
Speed/(cm/s)	2.8	2.8	2.8	2.8	2.8	2.8

**Table 4:** I_D_/I_G_ of each sample.

Name	Bare	pH = 6	pH = 5	pH = 4	pH = 3	pH = 2
I_D_/I_G_	1.008	0.979	0.976	0.947	0.901	0.937

**Table 5:** Atomic concentration table.

Name	Bare	pH = 6	pH = 5	pH = 4	pH = 3	pH = 2
C	76.5	81.2	81.5	81.79	82.28	78.18
O	23.5	18.8	18.5	18.21	17.72	21.82

**Table 6:** The initial oxidation temperature and the complete weightlessness temperature.

Name	Bare	pH = 6	pH = 5	pH = 4	pH = 3	pH = 2
Initial oxidation temperature/°C	540	610	626	681	696	661
Complete weightlessness temperature/°C	749	807	825	853	859	820

## Data Availability

The original contributions presented in this study are included in the article. Further inquiries can be directed at the corresponding author.

## Supplementary Materials

The following supporting information can be downloaded at: https://www.mdpi.com/article/10.3390/ma18174093/s1, Figure S1: The samples’ preparation progress by FIB: (a) carbon fiber; (b) cutting area; (c) vertical view of the cutting area with Pt protective layer; (d) front view of the cutting area with Pt protective layer; (e) carbon fiber cut; (f) the rest carbon fiber; (g) sample in low power; (h) sample in high power.
