# Supplementary material for: Preparation of a Novel Carbon Nano Coating on Carbon Fiber Surface Based on Plasma Electrolysis Effect
Source: Materials (Basel). 2025 Sep 1;18(17):4093. doi: 10.3390/ma18174093 (PMC12430531; doi:10.3390/ma18174093)
Supplement: Supplementary file 1 [file materials-18-04093-s001.zip › materials-3744833-supplementary.pdf]

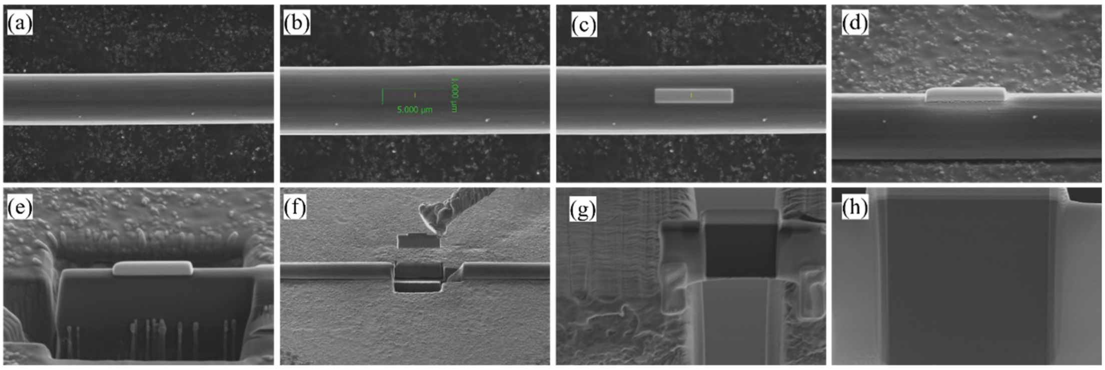

Figure S1. The samples' preparation progress by FIB: (**a**)carbon fiber; (**b**) cutting area; (**c**) vertical view of the cutting area with Pt protective layer; (**d**) front view of the cutting area with Pt protective layer; (**e**) carbon fiber cut; (**f**) the rest carbon fiber; (**g**) sample in low power; (**h**) sample in high power.
